# Chitosan oligosaccharide alleviates the growth inhibition caused by physcion and synergistically enhances resilience in maize seedlings

**DOI:** 10.1038/s41598-021-04153-3

**Published:** 2022-01-07

**Authors:** Jingchong Li, Aohui Han, Lei Zhang, Yang Meng, Li Xu, Feixiang Ma, Runqiang Liu

**Affiliations:** 1 Henan Engineering Research Center of Green Pesticide Creation & Intelligent Pesticide Residue Sensor Detection and School of Resources and Environment, Henan Institute of Science and Technology, Xinxiang, 453003 Henan China; 2 Present Address: Henan Institute of Science and Technology, Xinxiang, 453003 Henan China

**Keywords:** Oxidoreductases, Plant physiology, Leaf development

## Abstract

The use of biopesticides has gradually become essential to ensure food security and sustainable agricultural production. Nevertheless, the use of single biopesticides is frequently suboptimal in agricultural production given the diversity of biotic and abiotic stresses. The present study investigated the effects of two biopesticides, physcion and chitosan-oligosaccharide (COS), alone and in combination, on growth regulation and antioxidant potential of maize seedlings by seed coating. As suggested from the results, physcion significantly inhibited the growth of the shoots of maize seedlings due to the elevated respiration rate. However, COS significantly reduced the growth inhibition induced by physcion in maize seedlings by lowering the respiration rate and increasing the content of photosynthetic pigments and root vigor, which accounted for lower consumption of photosynthesis products, a higher photosynthetic rate and a greater nutrient absorption rate. Thus, an improved growth was identified. As indicated from the in-depth research, the application of physcion and COS combination is more effective in down-regulated the malondialdehyde (MDA) content by facilitating the activities of the antioxidative enzymes (i.e., superoxide dismutase (SOD), catalase (CAT) and guaiacol peroxidase (G-POD)). Such results indicated that the combined use of physcion and COS neither affected the normal growth of maize seedlings, but also synergistically improved the antioxidant potential of the maize plants, resulting in plants with high stress resistance. Thus, the combined use of physcion and COS by seed coating in maize production has great potential to ensure yield and sustainable production of maize.

## Introduction

Maize, one of the ‘Big Three’ cereal crops critical to human survival, provides over 30% of the heart demand for more than 4.5 billion people worldwide^1,2^. In addition, maize takes up a vital position in the feed and industry. For instance, ethanol produced from maize as a raw material is of high significance to the emerging biofuel field^3^. Since it shows high importance to the maintenance of an increasing population and industry, maize is critical to boosting the development of society and tackling down the problems regarding people’s livelihood. However, in maize production, the yield and quality of maize are extremely vulnerable to abiotic stress (e.g., salt, drought and pesticides)^4^ and biotic stress (e.g., diseases and pests)^5^.

Under abiotic stresses (e.g., salt, drought and cold), the redox homeostasis of plants is disturbed, which causes excessive accumulation of reactive oxygen species (ROS) in the plant cells and thus triggers oxidative stress^6^. The plant's enzymatic antioxidant system functions through the enzymatic reactions of antioxidant enzymes (e.g., SOD, CAT and POD) to maintain the normalization of intracellular ROS levels and thus guarantee the healthy growth of the plant^4^. Accordingly, high antioxidant enzyme activity is critical to the improvement of plant resistance to abiotic stresses.

With the advent of the green revolution, the application of chemical pesticides has been recognized as the primary strategy to protect maize crops from numerous biotic stresses, such as pests and diseases, thereby significantly increasing agricultural yields. However, the inappropriate application and overuse of such chemicals have triggered numerous problems (e.g., the emergence of resistance in target organisms, food contamination and environmental pollution)^7^. Furthermore, as the industry has been advancing, and the global population is rising, the area of arable land tends to decrease worldwide, while maize is increasingly demanded^8^. Accordingly, technologies are urgently required to maintain the green and sustainable production of maize. The existing technology exhibiting high potential is the application of biological pesticides for its advantages of low risk, low residue and environmental friendliness^9^. Furthermore, multiple biopesticides in combination may be a trend as the use of a single biopesticide is no longer sufficient to combat the diversity of biotic and abiotic stresses.

Chitosan oligosaccharide (COS) acts as a biogenic substance degradation from chitosan, and it exhibits a higher bio-activity than chitosan^10^. A previous study in our laboratory showed that COS was effective in promoting growth and antioxidant enzymes activities in wheat seedlings^11^. Meanwhile, previous reports have demonstrated that COS can improve salt tolerance in wheat seedlings^8^ and drought tolerance in maize plants^12^. The desirable effects of COS on plant growth have also been extensively reported over the past decade (e.g., stimulating the growth of plant tissue^13^, increasing the photosynthetic pigments, improving the photosynthesis efficiency and enlarging the chloroplast^14^, promoting nutriments uptake^15^). Furthermore, existing studies found COS to be capable of improving plants’ resistance to diseases by regulating various physiological processes. For instance, COS has been reported to be able to induce the innate immune response of oilseed rape and effectively reduce the infection of pathogenic bacteria^16^. Moreover, COS has also been suggested to increase the resistance to tobacco mosaic virus (TMV) in *Arabidopsis* by stimulating the salicylic acid signaling pathway^17^.

Physcion refers to a novel biological fungicide, extracted from the root of *Rheum palmatum L.*, a plant belonging to the family of polygonaceae^18^. It has been extensively found that physcion can be directly absorbed by plants to induce plants to produce defense responses, and it can inhibit the growth of pathogenic fungi, which protects crop plants from damage attributed to diseases^19^. In China, physcion has been widely adopted to prevent and treat powdery mildew, and it is termed as an effective cure for powdery mildew^18^. Furthermore, as demonstrated by existing studies, physcion significantly impacts other crop diseases. For instance, physcion was found to effectively reduce the damage of downy mildew to cucumber plants^20^ and gray mold to tomato plants^21^. Besides, physcion was indicated to effectively inhibit the growth of *Magnaporthe oryzae*^19^. Furthermore, physcion, a biological fungicide particularly suitable for crops, fruits, organic vegetables and others, is safe for humans and animals and exhibits environmental friendliness.

Since crops face a variety of biotic and abiotic stresses during growth, it is difficult to ensure high and stable crop yields by singularly improving the plant’s resilience or resistance to fungal diseases. Therefore, combinations that effectively improve disease resistance and stress tolerance of the plant, such as physcion and COS, are of great importance in ensuring food security. Various studies have established that COS can regulate plant growth and effectively improve plant tolerance to salt^8^ and drought^22^ stresses by increasing antioxidant enzyme activity. The excellent effectiveness of physcion against fungal diseases has also been documented. Nevertheless, it has not been reported that the combined use of two widely used biopesticides in agricultural production, physcion and COS, would have any effect on the growth, disease resistance and resilience of crop plants. Furthermore, the effect of physcion on plant growth and resilience has remained unrecorded. Hence, this study was conducted to investigate the effects of physcion and COS, alone and in combination, on the growth and antioxidant system of maize plants by seed coating. This study primarily aimed to study the effects of physcion alone and in combination with COS: (1) on the growth and development of maize seedlings via morphology, biomass accumulation and physiological variations, (2) on ROS levels, MDA content and antioxidant enzymes activities of maize seedlings, as well as (3) whether the combination of physcion and COS exerts a synergistic effect on oxidation resistance.

## Materials and methods

### Maize seeds and experimental drug source

Maize seeds of Zhengdan 958 cultivar provided by the Henan Academy of Agricultural Sciences (Zhengzhou, Henan, China). 98% Chitosan oligosaccharide sample provided by Ocean University of China (Qingdao, Shandong, China) and 0.8% physcion gel suspension agent originated from Beijing Kingbo Biotech Co., Ltd., (Beijing, China).

### Seed coating

The preparation of the seed coating formulations was technically supported by partner Beijing Kingbo Biotech Co., Ltd., (Beijing, China) and done in our laboratory. The COS concentrations used in the preparation of the seed coating formulations were obtained from preliminary experiments (see Supplementary Table S1 online). Preparation of COS coating formulation: 200 mg COS was dissolved in 1 L of distilled water, and subsequently 2 g of polyvinyl alcohol and 3 g of cellulose were added to the solution as a COS coating formulation. The 0.8% physcion gel suspension agent was adopted as the formulation of physcion seeds coating. Preparation of COS and physcion coated compositions: the combination formulation of physcion and COS was prepared by dissolving 200 mg COS in 1 L of 0.8% physcion suspension agent. Based on the registration of 0.8% physcion gel suspension on the China Pesticide Information Network, all the treatments were performed at 1:50 (formulation: seed, w/w). The control was treated with distilled water in the same proportion. The seed coating process was performed manually by mixing a fixed mass proportion of the coating material with the seeds, so the formulation is evenly covered with the seeds. The coated seeds were naturally dried before sowing.

### Plant materials and cultivate conditions

After being dried, the seeds were planted 15 per pot in plastic pots (30 cm in diameter and 29 cm high) filled with 6 kg of soil (1:1 w/w field soil/nutrient soil). Subsequently, the pots were maintained in a greenhouse at 30 ± 2 °C for the light stage and 25 ± 2 ℃ for the dark stage, with 75% relative humidity and 14 h light/10 h dark. After 21 days of the natural growth, the maize seedlings were collected from the whole plant to determine morphological and physiological parameters. Furthermore, the potted plants were grouped by random blocks, and each treatment group covered five biological replicates.

### Morphology and biomass

Five maize seedlings were randomly taken out from the five pots of the respective treatment group and then rinsed with tap water. Next, the roots and leaves of the seedlings were separated and then scanned with Epson Perfect V850 Photo Scanner (Epson, Inc., USA). Next, the WinRHIZO 2007 (Regent Instruments Inc, Canada) was adopted to calculate the leaf projection area, the total root length, the total root surface area, as well as the root average diameter. Before the scanning, the height of seedlings was examined.

Another five seedlings were used to determine biomass accumulation. The shoots and roots of maize seedlings were separated and then weighted as fresh weight. Afterward, the fresh samples were placed in an electric heating blast drying box (DHG-2200B, Zhengzhou Shengyuan instrument Co. LTD) at 105 ℃ for 30 min and then kept at 80 ℃ for 6 h. Then, the dry weight was measured.

### Photosynthetic pigment content and fluorescence characters

The ethanol extraction method was adopted to measure the content of chlorophyll a, chlorophyll b and carotene in this study. In brief, the 0.1 g chopped leaves were leached in a tube supplemented with 5 mL of 95% ethanol for 36 h in dark. Subsequently, the solution obtained with the leaching was measured at 665, 649 and 470 nm absorbance, respectively. The measured values were changed as the content of photosynthetic pigments by the formula reported in the existing study^23^, expressed in mg/g FW (fresh weight).

With a chlorophyll fluorescence system, the chlorophyll fluorescence parameters were detected (PEA, Hansatech Instruments Ltd, King’s Lynn, UK). After the dark adaptation was conducted for 30 min, the chlorophyll fluorescence parameters were measured.

### Root vigor

This study employed the method reported by Fontana et al.^24^ to measure the root vigor. Dehydrogenase could reduce triphenyltetrazolium chloride (TTC) to red triphenyltetrazolium (TTF), which altered the color of the root. In brief, 0.5 g root samples were cut into 1 cm length and then placed in a tube with 5 mL 0.6% TTC. Next, the tubes were incubated for 24 h in darkness at 30 °C, Then, the TTC solution was poured out and rinsed 4–5 times to remove the residue. Subsequently, 5 mL of 95% ethanol was added to the tube and then boiled for 10 min to extract TTF from the roots. The final solution absorbance was monitored at 485 nm. The root vigor was defined as OD/g FW (fresh weight).

### Respiration rate

The respiration rate was determined with polarographic O_2_ electrode-Chlorolab 2 (Hansatech Instruments Ltd, England) to measure the change in O_2_ concentration in a saturated CaSO_4_ solution. The monitoring lasted for 5 min, and the respiration rate was expressed as nM O_2_/min/g FW.

### Lipid peroxidation and relative electrolyte leakage

This study adopted the thiobarbituric acid (TBA) method to determine the MDA content in plant cells. The 0.1 g fresh samples were ground into powder under liquid nitrogen and then suspended in 1 mL 10% trichloroacetic acid (TCA) solution. Next, the samples were centrifuged at 10000 g with 4 ℃ for 15 min, and the supernatant was collected. Subsequently, the mixture of 0.5 mL of supernatant and 0.5 mL 10% TCA was boiled for 20 min, and the temperature rapidly cooled down with ice. The boiled mixture was centrifuged at 10,000*g* with 4 ℃ for 10 min, and then the supernatant was collected. Furthermore, the final supernatant absorbance was monitored at 450 nm, 532 nm and 600 nm, respectively, and the MDA content was determined as nmol/g FW (fresh weight) by adopting the formula of the existing study^25^.

Relative electrolyte leakage was assessed according to the existing method^26^. The 0.2 g root or leaf samples were cut with a scissor and then soaked in 10 mL of deionized water at 35 ℃ for 2 h. Next, the electrical conductivity of the solution (Lt) was determined. Subsequently, the samples were boiled in a water bath for 30 min, and the final electrical conductivity (L_0_) was measured after cooling to 25 ℃. The relative electrolyte leakage was defined as (L_t_/L_0_) × 100.

### ROS determination

The ROS level was determined by the superoxide anion (O_2_^·−^) and hydrogen peroxide (H_2_O_2_) levels in plant cells. The method proposed by Elstner et al.^27^ was employed to measure O_2_^·−^ level. The 0.1 g fresh sample was ground into powder under liquid nitrogen and suspended in 1 mL of 50 mM phosphate buffered saline (PBS). Then, the suspension was centrifuged at 10,000*g* with 4 ℃ for 20 min, and the supernatant was collected for the subsequent determination. Initially, a mixture of 0.05 mL of 6.25 mM PBS, 0.1 mL of 0.25 mM hydroxylamine hydrochloride and 0.05 mL of supernatant was incubated for 1 h at 25 ℃ in darkness. Afterward, 0.1 mL of 4.25 mM p-aminobenzenesulfonic acid and 0.1 mL of 1.25 mM 〈-naphthylamine were introduced to the initial mixture and then incubated under identical conditions for another 20 min. The absorbance of the final mixture was monitored at 530 nm, and the value was changed to O_2_^·−^ concentration, as expressed by nmol/g FW (fresh weight). The standard was prepared with NaNO_2_.

The H_2_O_2_ level was determined by measuring the absorbance of the purple complex formed by Fe^3+^ and dimethylphenol orange^28^. The 0.1 g fresh sample was ground into powder under liquid nitrogen. Then, the powders were suspended in 1 mL of cold acetone and then centrifuged at 10,000*g* with 4 ℃ for 15 min, and the supernatant was collected. The absorbance of the mixture of 0.2 mL of distilled water, 0.3 mL of supernatant, 0.5 mL of 400 mM sorbitol, 0.5 mL of 0.8 mM ferrous ammonium sulfate and 0.5 mL of 0.4 mM dimethylphenol orange was monitored at 560 nm after the mixture was incubated at 30 ℃ for 30 min. The standard was prepared with a diluted 30% H_2_O_2_.

### Activities of antioxidant enzymes

The enzyme was extracted according to the method reported by Zhang et al.^6^. 0.5 g of the samples were ground into powder under liquid nitrogen. The powders were suspended in 5 mL phosphate buffer (100 Mm, pH 7.4) supplemented with 1% polyvinylpyrrolidone (PVP) and then centrifuged at 12,000*g* with 4 ℃ for 20 min. Next, the supernatant was collected.

The nitrogen blue tetrazolium method was used to determine SOD (EC 1.15.1.1) activity^29^. SOD could inhibit the reaction between riboflavin and NBT, and the activity of SOD was estimated by measuring the absorbance at 560 nm after the 40 min incubation at 37 ℃ with light. One enzyme unit was defined as the amount of SOD required to achieve 50% inhibition in a 1 mL reaction mixture.

The CAT (EC1.11.1.6) activity was assayed with the method proposed by Chen et al.^30^. In this case, 20 µL samples of the enzyme extract were added to 4.98 mL of 100 mM phosphate buffer (pH 7.0) supplemented with 50 mM H_2_O_2_, and the enzyme activity was measured by complying with the change in absorbance of H_2_O_2_ at 240 nm over a 1 min period.

The G-POD (EC1.11.1.7) activity was assessed with the guaiacol method^31^, which was achieved by mixing 50 µL enzyme extract with 4.5 mL of 100 mM phosphate buffer (pH 6.0) supplemented with 50 mM H_2_O_2_ and 75 mM guaiacol. Based on the variation in absorbance of the mixture at 470 nm over a 1 min period, the POD activity was measured.

The CAT and G-POD activity was represented by the fresh weight (FW), and one unit of enzyme activity was defined as 0.01 unit of the OD change per min, expressed as unit /mg FW.

### Statistical analysis

The biomass and morphology tests were performed in quintuplicate, and the remaining tests were performed in triplicate. SPSS software package (ver. 22.0; SPSS Inc., Chicago) was adopted for the one-way analysis of variance (ANOVA), and statistical differences between treatments were conducted by performing Duncan's multiple range test (*P* < 0.05).

### Ethics declarations

We had obtained permission to collect plants. All experiments were performed following the relevant guidelines/regulations.

## Results

### Effect of combined application of physcion and COS on root and leaf development of maize

**Figure 1:**
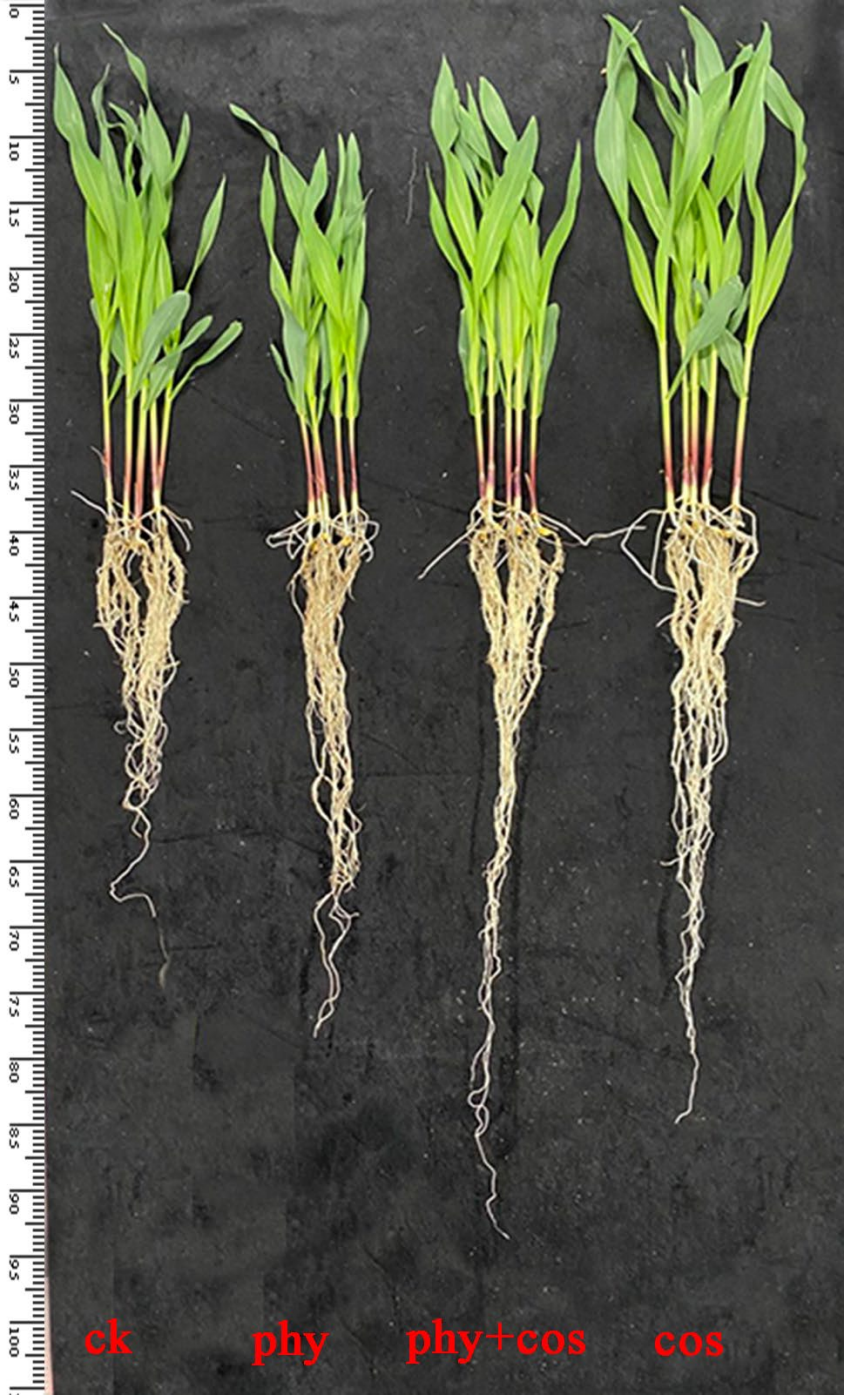
Effects of physcion and COS, alone or in combination, on development and growth of maize seedlings. Phy represent physcion. Phy + cos represent the combination of physcion and COS.

Morphological parameters acted as vital indicators to assess the effects of a new substance on plant growth. As suggested from the results of this study, both the single use of physcion and COS or in combination significantly affected the growth of maize seedlings (Fig. 1).

The data revealed that separate treatments of physcion and COS caused opposite effects on shoots growth of maize seedlings. Physcion significantly (*P* < 0.05) inhibited the development of shoots, which was manifested as a decrease of 19.36% in the plant height and 26.35% in the projected leaf area in comparison with the control (Table 1). However, COS significantly promoted maize shoots development, with plant height and leaf area increasing by 7.02% and 15.97%, respectively, as compared to the control. Unsurprisingly, COS significantly alleviated the inhibition of stem growth caused by physcion. Compared with physcion alone, the combined use of physcion and COS increased the plant height and the leaf projection area by 15.32% and 14.74%, respectively. In addition, the plant height and leaf area of the combined physcion and COS-treated maize seedlings were inferior to the COS-treated group due to the inhibitory effect of physcion on the growth of shoots.

**Table 1:** Effects of physcion and COS, alone or in combination, on root and leaf development of maize seedlings^Z^.

Treatment	Plant height (cm)	Projected leaf area (cm^2^)	Total root length (cm)	Total root surface area (cm^2^)	Average root diameter (mm)
CK	33.04 ± 0.40b	64.45 ± 1.37b	1171.17 ± 11.85c	130.16 ± 1.83d	0.329 ± 0.004d
Physcion	27.68 ± 0.49c	51.01 ± 0.73d	1315.04 ± 8.98b	152.65 ± 1.35c	0.381 ± 0.006b
COS	35.36 ± 0.57a	74.74 ± 0.76a	1415.41 ± 11.67a	162.42 ± 2.02b	0.360 ± 0.001c
Physcion + COS	31.92 ± 0.36b	58.53 ± 1.04c	1425.37 ± 8.58a	176.22 ± 2.41a	0.413 ± 0.006a

^Z^Data are means ± standard error of five independent experiments with two replicates in each experiment. The values followed by different letters within the same column are significantly different at P < 0.05 significance level according to the Duncan’s multiple ranges test. physcion + COS represent the combination of physcion and COS.

It is noteworthy that physcion and COS significantly (*P* < 0.05) facilitated the development of the root. With physcion and COS treatments, the total root length, the root surface area and the average root diameter increased by 12.28%, 17.28%,15.81% and by 20.85%, 24.78%, 9.42%, respectively, as compared to the control (Table 1). Furthermore, the combined use of physcion and COS showed better facilitation than physcion alone, and the total root length, the surface area and the average root diameter increased by 8.39%, 15.44% and 8.40%, compared with those achieved by using physcion alone. Although both the total root surface area and average root diameter of the combined physcion and COS-treated maize seedlings were significantly higher than those of the COS-treated group, there was no significant difference in the total root length between both groups.

### Effect of combined application of physcion and COS on biomass accumulation of maize

Not surprisingly, both the effects of physcion and COS on biomass accumulation and root and shoot development display a similar trend. Physcion alone significantly inhibited the accumulation of shoots biomass with the fresh and dry weight of shoots was reduced by 32.95% and 33.67% as compared with the control (Table 2). And COS alone significantly promoted the accumulation of shoots biomass with the fresh and dry weight of shoots was increased by 32.65% and 24.29% as compared with the control. Meanwhile, physcion and COS alone significantly (*P* < 0.05) increased the accumulation of the roots biomass, the fresh and dry weight of the roots increased by 21.46%, 22.70% and by 37.29, 36.79%, respectively, when compared with the control. Furthermore, the combination of physcion and COS reduced the inhibited effect on shoots biomass accumulation induced by physcion, and further facilitated the accumulation of the roots biomass. Compared with physcion alone, the combination of physcion and COS significantly increased the biomass accumulation of maize seedlings, with the shoot fresh and the dry weight increasing by 31.15% and 31.62%, respectively, and with the root fresh and the dry weight increasing by 12.23% and 8.45%, respectively (Table 2). However, the dry and fresh weights of shoots of maize seedlings from the combined physcion and COS treatments were significantly lower than those from the COS treatment, but there was no difference in the dry and fresh weights of the roots between the two treatments.

**Table 2:** Effects of physcion and COS, alone or in combination, on biomass accumulation of maize seedlings^Z^.

Treatment	Shoot fresh weight (mg)	Root fresh weight (mg)	Shoot dry weight (mg)	Root dry weight (mg)
CK	2250.0 ± 37.0b	1245.0 ± 15.8c	155.6 ± 2.1b	102.2 ± 3.3c
Physcion	1692.4 ± 12.3c	1512.2 ± 27.4b	116.4 ± 3.0c	125.4 ± 2.5b
COS	2984.6 ± 19.7a	1709.2 ± 15.9a	193.4 ± 8.3a	139.8 ± 1.7a
Physcion + COS	2219.6 ± 31.5b	1727.4 ± 16.9a	153.2 ± 2.9b	136.0 ± 4.6a

^Z^Data are means ± standard error of five independent experiments with two replicates in each experiment. The values followed by different letters within the same column are significantly different at P < 0.05 significance level according to the Duncan’s multiple ranges test. physcion + COS represent the combination of physcion and COS.

### Effect of combined application of physcion and COS on the content of photosynthetic pigments and fluorescence characters of maize

The growth and development of plants are largely determined by the carbon assimilation of photosynthesis, and the photosynthetic efficiency is determined by the content of photosynthetic pigments. As indicated from the data in this study, physcion alone did not impact the chlorophyll and carotene contents, as well as Fv/Fm (Table 3). However, COS alone and physcion combined with COS significantly (*P* < 0.05) increased the contents of photosynthesis pigments with the total chlorophyll content in contrast with the control increasing by 14.62% and 6.60%, respectively, and carotene content increased by 21.62% and 13.51%, respectively (Table 3). Meanwhile, COS alone and physcion combined with COS significantly promoted Fv/Fm. In addition, physcion combined with COS significantly increased photosynthetic pigment content and Fv/Fm of maize seedlings, as compared with physcion treatment. However, physcion combined with COS treatment was even slightly inferior to COS according to photosynthetic pigment content and Fv/Fm.

**Table 3:** Effects of physcion and COS, alone or in combination, on photosynthetic pigments content and fluorescence characters of maize seedlings^Z^.

Treatment	Chlorophyll a (µg/g)	Chlorophyll b (µg/g)	Carotene (µg/g)	Total chlorophyll (µg/g)	Fv/Fm
CK	6.65 ± 0.04c	2.58 ± 0.04c	1.11 ± 0.01b	9.23 ± 0.03c	0.71 ± 0.00b
Physcion	6.62 ± 0.03c	2.56 ± 0.03c	1.11 ± 0.03b	9.19 ± 0.03c	0.71 ± 0.00b
COS	7.56 ± 0.09a	3.02 ± 0.05a	1.35 ± 0.03a	10.58 ± 0.04a	0.73 ± 0.00a
Physcion + COS	7.11 ± 0.04b	2.73 ± 0.05b	1.26 ± 0.04a	9.84 ± 0.02b	0.72 ± 0.00a

^Z^Data are means ± standard error of five independent experiments with two replicates in each experiment. The values followed by different letters within the same column are significantly different at P < 0.05 significance level according to the Duncan’s multiple ranges test. physcion + COS represent the combination of physcion and COS.

### Effect of combined application of physcion and COS on the root vigor and plant respiration rate of maize

The effects of physcion and COS on the root vigor were also investigated. As revealed from the data, the use of physcion, COS alone or in a combination of both significantly (*P* < 0.05) increased the root vigor by 26.92%, 40.38% and 51.54% in contrast with the control, respectively (Table 4). In addition, the combination of physcion and COS demonstrates a potential synergy in promoting the root vigor.

**Table 4:** Effects of physcion and COS, alone or in combination, on plant respiration reflected by oxygen consuming rate and root vigor of maize seedlings^Z^.

Treatment	Root vigor (OD/FW)	Leaf	Root
		O_2_ consuming rate (nM O_2_/min/g FW)	
CK	2.60 ± 0.10c	8.22 ± 0.22b	14.48 ± 0.58c
Physcion	3.30 ± 0.15b	13.55 ± 1.64a	21.32 ± 1.05a
COS	3.65 ± 0.14ab	8.32 ± 0.53b	15.36 ± 0.20bc
Physcion + COS	3.94 ± 0.07a	10.36 ± 0.73b	18.04 ± 0.57b

^Z^Data are means ± standard error of five independent experiments with two replicates in each experiment. The values followed by different letters within the same column are significantly different at P < 0.05 significance level according to the Duncan’s multiple ranges test. physcion + COS represent the combination of physcion and COS.

Physcion exerted a similar effect on plant respiration rate with the root vigor and significantly elevated the plant respiration rate, with the leaf and root respiration rate increased by 64.84% and 47.24%, respectively, as compared with the control (Table 4). However, the combination of physcion and COS significantly decreased the plant respiration rate; to be specific, the leaf and root respiration rates decreased by 30.79% and 18.18%, respectively, as compared with those achieved by using physcion alone (Table 4). However, the respiration rate of seedlings in the combined physcion and COS treatment group was still higher than that of the control group. Furthermore, the respiration rate of physcion + COS treatment was slightly higher than that of COS treatment, but not significantly different. It is noteworthy that COS did not obviously impact the plant respiration rate.

### Effect of combined application of physcion and COS on lipid peroxidation and cell membrane damage of maize

**Figure 2:**
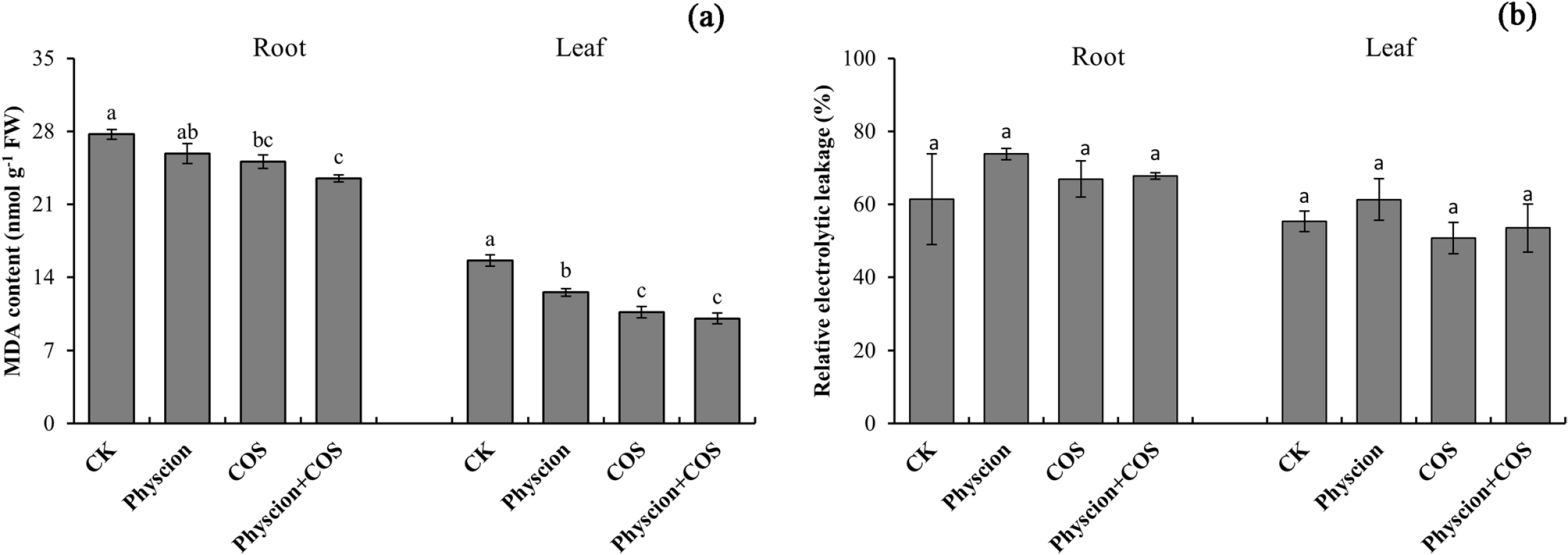
Effects of physcion and COS, alone or in combination, on MDA content (**a**) and relatively electrolytic leakage (**b**) for leaves and roots of maize seedlings. Different lowercase letters indicate significant difference (P < 0.05). Error bars indicate standard errors calculated for three replications. Physcion + COS represent the combination of physcion and COS.

The MDA content, an indicator of cytomembrane peroxidation, could provide significant insights into the degree of oxidative stress experienced by plant cells^32^. Given the different effects exerted by physcion and COS on maize seedling shoots and roots, the MDA content in leaves and roots was investigated together. As revealed from the data, physcion, COS alone and physcion combined with COS significantly (*P* < 0.05) decreased MDA content as compared with the control with decreased by 7.15%, 10.43% and 18.07% in leaves and 23.95%, 45.71% and 54.76% in the roots, respectively (Fig. 2a). In addition, the combination of physcion and COS was more effective in inhibiting the accumulation of MDA in maize tissues than both of them alone. Compared to physcion and COS alone, the MDA content was reduced by 10.19% and 6.91% in leaves and 24.85% and 6.21% in the roots, respectively.

Relative electrolytic leakage acts as a reliable indicator of the degree of cell membrane damage. In this study, the relative electrolytic leakage was also investigated. However, as revealed from the data, physcion, COS alone and physcion combined with COS did not exert a significant effect on root relative electrolytic leakage, even though the root relative electrolytic leakage increased by 20.19%, 9.00% and 10.44%, respectively, as compared with the control (Fig. 2b). In addition, COS alone and physcion combined with COS reduced the leaf relative electrolyte leakage by 8.88% and 3.33%, respectively, as compared with the control. Likewise, there were no statistical differences in the treatment groups and between treatment and control.

### Effect of combined application of physcion and COS on ROS level of maize

**Figure 3:**
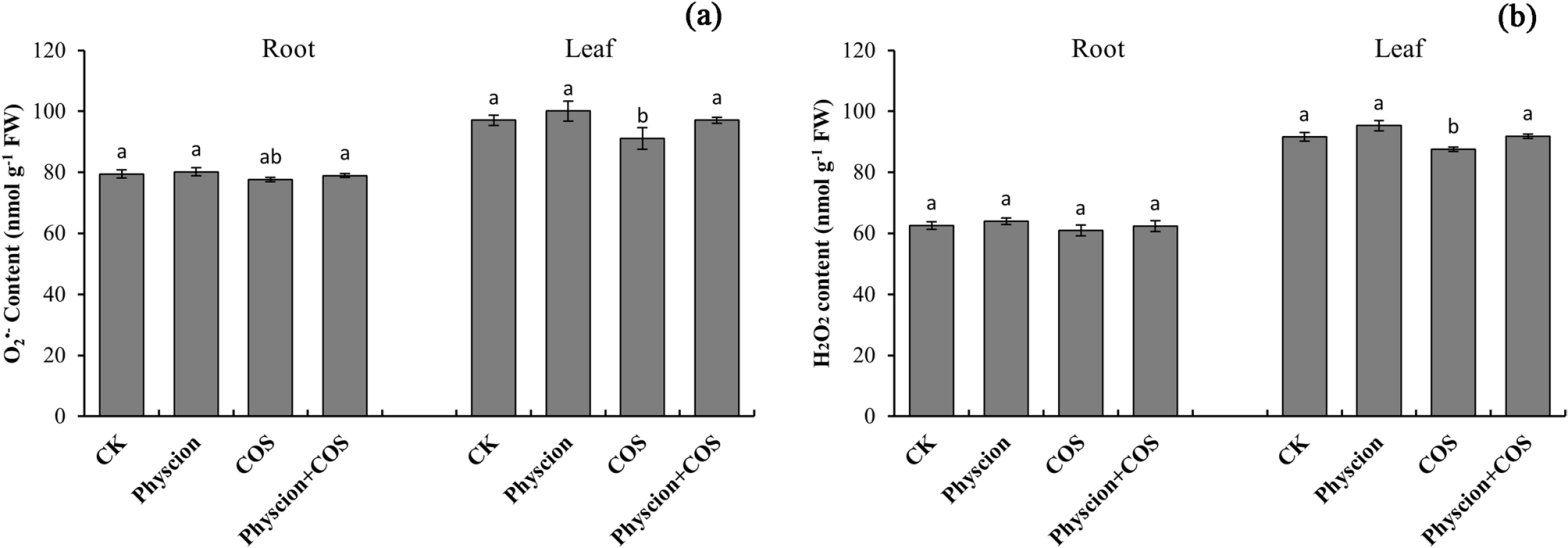
Effects of physcion and COS, alone or in combination, on O_2_^·−^ (**a**) and H_2_O_2_ (**b**) levels in leaves and roots of maize seedlings. Different lowercase letters indicate significant difference (P < 0.05). Error bars indicate standard errors calculated for three replications. Physcion + COS represent the combination of physcion and COS.

The ROS level was measured by the levels of O_2_^·−^ and H_2_O_2_ in this work. As suggested from the results, COS significantly (*P* < 0.05) reduced the O_2_^·−^ content in leaves and roots with reduced by 2.27% and 6.54%, respectively, as compared with the control (Fig. 3a). However, physcion alone and physcion combined with COS have not an obvious effect on leaf and root O_2_^·−^ content, and there was no statistical difference compared with the control.

Different from the O_2_^·−^, COS significantly reduced the H_2_O_2_ content in the leaves by 4.56% compared to the control; however, there was no noticeable effect on the H_2_O_2_ content in the roots (Fig. 3b). Furthermore, physcion alone and physcion combined with COS have not a significant effect on both leaf and root H_2_O_2_ content. Likewise, there was no statistical difference between the combination of physcion and COS compared to its use alone.

### Effect of combined application of physcion and COS on antioxidant enzymes activities of maize

**Figure 4:**
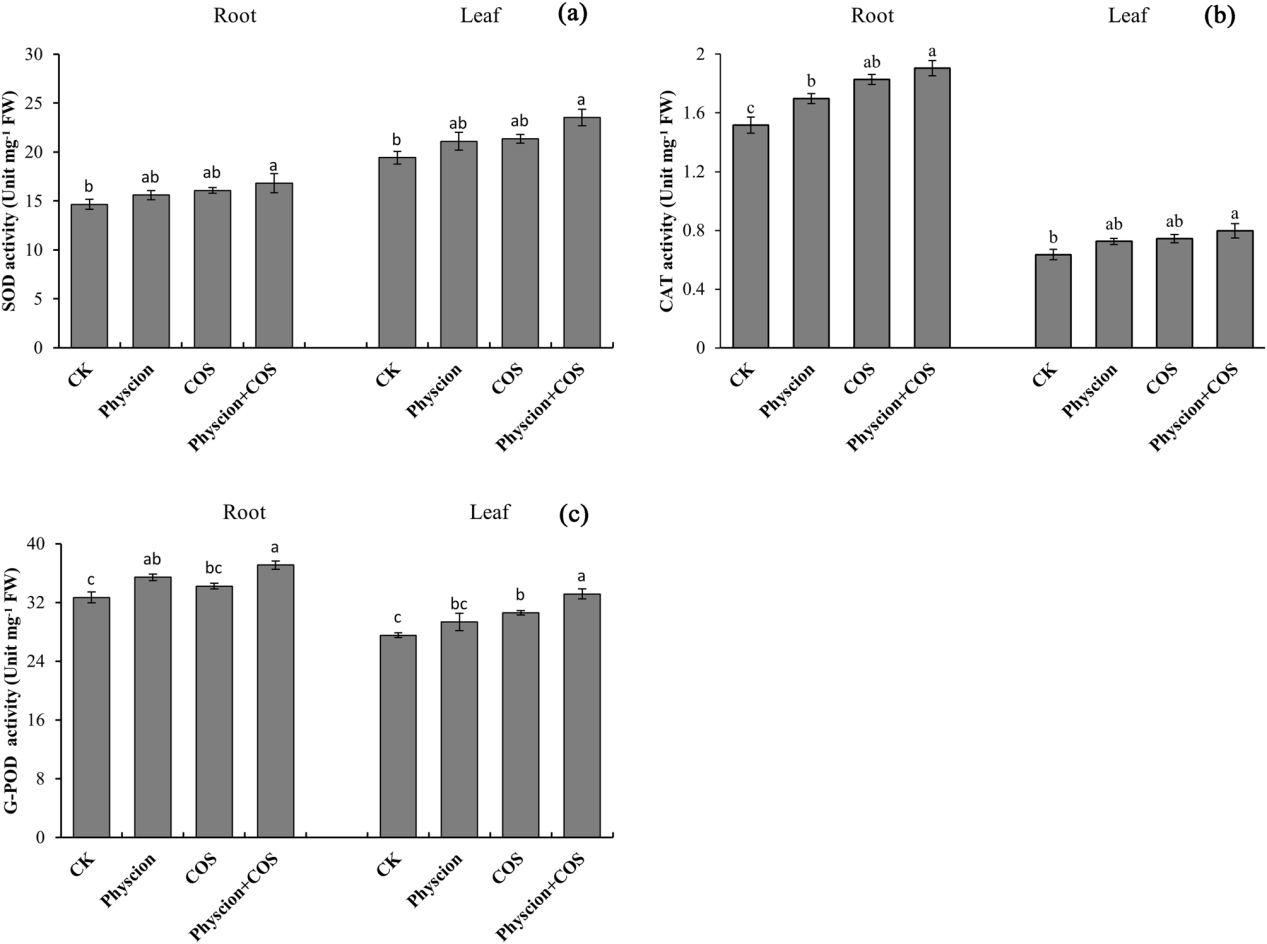
Effects of physcion and COS, alone or in combination, on superoxide dismutase (**a**), catalase (**b**), guaiacol peroxidase (**c**), activities in leaves and roots of maize seedlings. Different lowercase letters indicate significant difference (P < 0.05). Error bars indicate standard errors calculated for three replications. Physcion + COS represent the combination of physcion and COS.

The antioxidant enzymes activities have been investigated as well. As suggested from the results, physcion, COS and physcion combined with COS significantly (*P* < 0.05) promoted SOD activity by 6.46%, 9.70% and 14.72% in the root, and by 8.65%, 9.95% and 21.18% in leaves, as compared with the control (Fig. 4a). Furthermore, the combination of physcion and COS showed better SOD activity enhancement, with the SOD activity of the roots increasing by 7.76% and 4.57%, and that of leaves increasing by 11.53% and 10.21%, respectively, as compared to physcion and COS alone.

Similar to SOD, the physcion, COS and physcion combined with COS also pronounced promoted CAT activity as compared with the control, with the increase in the root CAT activity by 12.02%, 20.57% and 25.58%, and with the increase in the leaf CAT activity by 14.23%, 17.17% and 25.79% (Fig. 4b). As expected, the use of physcion combined with COS also exerted a better promotion effect on the CAT activity, with the CAT activity of the roots increasing by 12.11% and 4.16%, and that of leaves increasing by 10.11% and 7.35%, respectively, as compared with physcion and COS alone.

No surprise, the physcion, COS and physcion combined with COS also pronounced promoted G-POD activity as compared with the control, with the increase in the root G-POD activity by 8.36%, 4.72% and 13.42%, and with the increase in the leaf G-POD activity by 6.55%, 11.07% and 20.41% (Fig. 4c). Likewise, a more effective promotion was exerted on the G-POD activity. The root G-POD activity of physcion combined with COS treated seedlings increased by 4.66% and 8.31% and leaf G-POD activity increased by 13.00% and 8.40%, respectively, compared with the treatments alone and COS.

## Discussion

Pesticides (e.g., insecticides, fungicides and herbicides) can importantly guarantee a high and stable production of crops yield^33^. However, the long-term unreasonable use of such chemicals has led to residues, increased resistance, and harm to non-target organisms. Meanwhile, the negative effects of pesticides on crop plant growth and development have been well documented by existing studies^34^. As a biological pesticide, physcion has been proven to inhibit the diseases attributed to fungi. However, its effect on the growth of crop plants (e.g., maize) has been rarely reported. Also, since maize is subject to multiple diseases and abiotic stresses during growth, it is difficult to achieve green and sustainable production of maize by employing single biopesticide. Accordingly, the combined use of physcion and COS may be more beneficial for maize production.

This study investigated the effects of physcion on maize seedlings. According to the results, exposure of physcion significantly inhibited maize seedlings’ shoot growth, as evidenced by the significant (*P* < 0.05) decrease in the plant height, the leaf projected area, and the fresh and dry weight of the shoots (Tables 1, 2). However, physcion significantly promoted the root development of maize seedlings, as manifested by the increase in the total root length, the total root surface area and volume, as well as the average root diameter (Table 1). The main reason for such a difference in growth might be the increase in plant respiration rate and root vigor. The relationship between respiration rate and plant growth has been effectively documented^35^. Though plants require the energy produced by aerobic respiration to absorb, transport and metabolize activities, an excessively high respiration rate would inevitably lead to excessive consumption of photosynthetic products, instead of being used for the plant's growth^36^. Furthermore, the high root vigor and respiration attributed to physcion lead to high root physiological activity that can promote the development of the roots^24^. In the absence of an increase in photosynthetic efficiency, an excessively high respiration rate and too fast root development inevitably led to the inhibition of shoots development. Though a well-developed root system could help the absorption of nutrients, it was suggested not to be enough to remedy the excessive consumption attributed to physcion.

This study, however, found that COS can significantly alleviate the physcion inhibition of shoot growth and further promote root development (Table 1). Physcion combined with COS significantly increased the plant height, total root length and surface area, as well as the fresh and dry mass of the shoots and roots compared with physcion alone (Tables 1, 2). Such remission might be explained as COS reduced the respiration rate of maize plants, so more photosynthesis products were used for the growth of the plant itself. In addition, as suggested from the results, physcion combined with COS also significantly increased the photosynthetic pigment content of maize plants compared with the control or physcion single treatment. The increase in the photosynthetic pigment content would inevitably elevate the photosynthetic rate, and plants usually relied on photosynthesis for carbon assimilation to maintain growth^37^, so the increase in the photosynthetic pigment content might be another vital reason for reducing shoots growth inhibition. This hypothesis was consistent with an existing study that also found COS significantly increased contents of chlorophyll in leaves of wheat plants were correlated with increases in the plant height, root length and biomass^38^. Indeed, similar results were also documented in several other crops (e.g., cotton^39^, soybean^40^, rice^41^ and pepper^42^).

Reactive oxygen species (ROS) is critical to maintaining and regulating plant growth^43^. However, excessive accumulation of ROS will cause oxidative stress that can destroy cell membrane structure, thereby affecting the normal growth and development of plants^34,44^. Existing studies reported that chemical pesticides induced over-accumulation of ROS in plant cells which eventually caused severe damage in growth, nutrient assimilation, photosynthesis, etc.^45–47^. However, the data of this study showed that physcion has no significant effect on the level of ROS in the root and leaf cells of maize seedlings (Fig. 2). This result showed that the use of biological pesticide physcion does not produce oxidative stress on plants like chemical pesticides. In addition, the beneficial effects of biological pesticide COS on plants have been well demonstrated^48,49^. Thus, as we expected, the combined application of physcion and COS did not increase the level of ROS in plant cells (Fig. 2). In addition, the data of this study showed that both physcion and COS used alone or in combination significantly reduced the MDA content in the root and leaf cells of maize plants (Fig. 1). MDA, one of the critical products of membrane lipid peroxidation, causes the cross-linking and polymerization of life macromolecules (e.g., proteins and nucleic acids), while exhibiting cytotoxicity^50^. Accordingly, the reduction of MDA content in this study indirectly indicates that physcion alone and in combination with COS did not negatively affect the integrity of cell membranes and improved the stress resistance of maize plants. Furthermore, the obvious synergistic effect on inhibiting the accumulation of MDA is worth special attention when physcion and COS are used together.

It is generally known that antioxidant enzymes (e.g., SOD, CAT and G-POD) play a critical role in eliminating excessive ROS accumulation of cells induced by abiotic and biotic stresses as well as in plant defense systems^51^. It is speculated that physcion alone or combined with COS may cause antioxidant enzymes activities to vary. For this reason, it is noteworthy that this study reported the significantly increased activities of SOD, G-POD and CAT were in the roots and leaves of maize seedlings after treatments with physcion alone or in combination with COS (Fig. 3). Given that the antioxidant enzymes activities are significantly closely linked to the capacity for plants to eliminate oxidative stress caused by stress, it might expect that physcion alone or combined with COS would improve the stress resistance of the maize plants. This conclusion was also confirmed by the current study found a significant (*P* < 0.05) reduction in the accumulation of MDA. Taken together, the mentioned results indicated that physcion alone or in combination with COS can significantly improve the tolerance of maize plants to adverse environmental factors. Also, the combined use of physcion and COS has a potential synergistic effect in increasing antioxidant enzyme activity in maize plants.

## Conclusions

In conclusion, the results of this study revealed that physcion adversely affected shoots growth of maize seedlings. However, COS significantly reduced physcion-induced growth inhibition in maize seedlings not only by decreasing the respiration rate, but also by increasing the content of photosynthetic pigments and root vigor. In addition, it was also reported that physcion alone and in combination with COS significantly improved the antioxidant enzyme activity, and reduced the MDA content. The noteworthy outcome of this study was that the combination with COS exerts a more desirable effect on antioxidant enzymes activities, which helps strengthen the antioxidant capacity of the plant and thus enhances resilience. However, further studies should be conducted to determine the effectiveness of the combination of physcion and COS to respond to fungal diseases. Furthermore, verification that the desired effects found to persist into the maturity of the maize and increase yields in the field also was the next focus.

## Supplementary Information

Supplementary Information.

## Data Availability

All relevant data are within the paper.
