# Supplementary material for: Chitosan oligosaccharide alleviates the growth inhibition caused by physcion and synergistically enhances resilience in maize seedlings
Source: Sci Rep. 2022 Jan 7;12:162. doi: 10.1038/s41598-021-04153-3 (PMC8742106; doi:10.1038/s41598-021-04153-3)
Supplement: Supplementary file 1 — Supplementary Information. [file 41598_2021_4153_MOESM1_ESM.pdf]

Potential synergistic effects of chitosan oligosaccharide and physcion on enhancing maize resilience

Jingchong Li[a, ‡], Aohui Han[a, ‡], Lei Zhang[a], Yang Meng[a], Li Xu[a], Feixiang Ma[a], Runqiang Liu[a, *]

Table S1. Biological characteristics of maize plants with increasing concentrations of chitosan oligosaccharide [Z]

Concentrations	Plant hight (cm)	Root length(cm)	Average aboveground portion of a single plant (g)		Average under-ground portion of a single plant (g)	
			Fresh weight	Dry weight	Fresh weight	Dry weight
0mg/L	37.4±2.0^{a}	8.1±0.8^{a}	2.200±0.143^{a}	0.188±0.014^{a}	0.468±0.016^{a}	0.029±0.002^{a}
25 mg/L	39.1±3.0^{ab}	8.4±0.8^{a}	2.356±0.136^{a}	0.194±0.016^{a}	0.476±0.025^{a}	0.032±0.003^{a}
50 mg/L	41.4±1.8^{ab}	9.6±0.7^{ab}	2.360±0.113^{a}	0.192±0.025^{a}	0.521±0.026^{ab}	0.040±0.002^{b}
100 mg/L	42.1±2.2^{ab}	9.5±0.9^{ab}	2.558±0.205^{ab}	0.210±0.011^{ab}	0.554±0.045^{b}	0.410±0.004^{b}
200 mg/L	44.9±1.3^{b}	11.5±0.6^{ab}	2.800±0.101^{b}	0.250±0.015^{b}	0.652±0.046^{b}	0.422±0.009^{b}
400 mg/L	43.8±2.8^{ab}	10.2±0.4^{b}	2.654±0.375^{ab}	0.235±0.011^{b}	0.601±0.037^{b}	0.415±0.002^{b}
800 mg/L	40.0±4.3^{ab}	9.8±1.0^{a}	2.421±0.048^{ab}	0.210±0.017^{ab}	0.485±0.064^{a}	0.038±0.002^{b}

[Z] Values represent the mean ± standard error for each parameter/concentration combination. Different letters represent significant differences for each treatment of different concentration of chitosan oligosaccharide at $\alpha = 0.05$.
